# Screening of Diabetes-Associated Autoantigens and Serum Antibody Profiles Using a Phage Display System

**DOI:** 10.1155/2024/1220644

**Published:** 2024-10-24

**Authors:** Jun Lin, Zhenyu Wang, Hongtao Wang, Yuping Li, Yao Liu, Yige He, Qian Liu, Zichuan Chen, Yuan Ji

**Affiliations:** ^1^Shenzhen Institute for Drug Control (Shenzhen Testing Center of Medical Devices), No. 28, Gaoxin Central 2nd Avenue, Nanshan, Shenzhen 518057, China; ^2^Shenzhen Blot Bio-Products Ltd, Nanshan Knowledge Service Building, 3025 Nanhai Avenue, Nanshan, Shenzhen 518052, China

**Keywords:** autoantibody, diabetes mellitus, epitope, phage display system

## Abstract

**Aims/Introduction:** Phage display method is a crucial tool to find novel clinically valuable diabetes-associated autoantigens and identify known autoantigen epitopes that are associated with diabetes and could provide scientific support and guidance for the artificial construction and synthesis of Type I diabetes mellitus (T1DM) novel biomarkers.

**Materials and Methods:** The phage display system was used for the “biopanning” of T1DM serum. Following the sequencing of the phage DNAs, the homologous sequences of the above fusion heptapeptide were further investigated by BLAST to track the origin of the polypeptide sequences. The antibody spectrum revealed new T1DM-associated epitopes and antibodies.

**Results:** A total of 1200 phage DNA were sequenced and 9 conserved polypeptide sequences were collected. It was confirmed that the zinc transporter and islet amyloid protease were among them. The conserved polypeptide sequence 8 and another three distinctive polypeptide sequences derived from Proteus were discovered. Furthermore, we expressed recombinant proteins with homologous polypeptide sequences for the human islet amyloid polypeptide (IAPP) and polypeptide precursor human zinc transporter 8 (ZNT8). Through clinical sample detection for the serum from T1DM (*n* = 100) and T2DM (*n* = 200) patients, results demonstrate the importance and relevance of these polypeptides in the recognition and classification of various forms of diabetes.

**Conclusion:** Human pancreatic and concurrent bacterial-derived protein antigens and their epitopes were identified in this research by the phage display system, which is crucial for distinguishing different types of diabetes.

## 1. Introduction

Diabetes is a clinical syndrome caused by an absolute or relative deficiency of insulin, which is related to the genetic background of individuals [1]. Diabetes can be classified into two categories [2], Type I (T1DM, IDDM, insulin-dependent diabetes mellitus) and Type II diabetes (T2DM, NIDDM, noninsulin-dependent diabetes mellitus). T1DM is featured by insulin insufficiency caused by islet *β*-cell destruction [3]. T1DM can be divided into immune-mediated diabetes and idiopathic diabetes [4]. Moreover, T2DM is predominantly caused by low insulin resistance and insulin insufficiency [5].

At present, the data about islet cell autoantigen corresponding to autoantibodies remain scarce. Autoantigens are still less understood in terms of their type, nature, origin, and makeup. In addition, scientists are of great interest for the nature of the antigen epitope that can react with autoantibody. Meanwhile, standardized autoantigens with high sensitivity toward autoantibodies are essential for the diagnosis and typing of diabetes, especially for autoimmune-associated T1DM. To study the nature of autoantigens and epitopes and to discover more autoantibodies in diabetes patients will help us understand the pathogenesis of T1DM and some autoimmune-associated T2DM. Moreover, the identification of specific and highly effective antigens can help the detection of autoantibodies in diabetes [6]. Therefore, the development of raw autoantigen materials is crucial.

In essence, the antigen-antibody interaction is an interaction between proteins. The phage random peptide display system is an important research tool developed in recent years for in vitro investigation of protein interactions [7]. Through the gene recombination of the random peptide coding sequence and phage minor coat protein pIII's sequence, the phage will express the random peptides as N-terminal fusions to the minor coat protein pIII [8]. By immobilizing target proteins on solid supports, the phage display technology accomplishes high-throughput reactive protein screening using biological screening techniques known as “biopanning” in vitro. Typically, after several rounds of screening/amplification, monoclonal phages that can react with the protein of interest can be obtained. Based on the above properties, phage display systems are suitable for identifying new ligands (such as receptor agonists) or for exhibiting interactions between two known proteins (such as studies of antibody epitopes) [9]. The aim of our experiment was to find phages that are only attracted to T1DM-related antibodies. To achieve this, we initially selected out phages that bind to immunoglobulins in the serum from healthy people and T2DM patients. Among the remaining phages, we select phages that have an affinity toward immunoglobulins in T1DM patients' serum. These phages were then reacted with T1DM serum multiple times to filter out those with low affinity against T1DM patients' serum. Results were confirmed by the clinical samples.

Our study has successfully examined the association between T1DM-associated autoantibodies and the relevant autoantigens using the phage display technology and also discovered two new epitopes against T1DM-associated autoantibodies.

## 2. Materials and Methods

### 2.1. Patients and Control Data

A total of 500 serum samples were obtained from the clinical lab center, Guangdong Provincial Hospital of Chinese Medicine, containing 100 T1DM patients, 200 T2DM patients, and the normal control group consisted of 200 healthy individuals. All of the diabetes serums mentioned above meet the WHO diagnostic criteria for diabetes. The subtypes (T1DM or T2DM) were confirmed and classified through laboratory tests and clinical symptoms. Patient demographics are shown in Table 1. The study was approved by the ethics committee of the Guangdong Provincial Hospital of Chinese Medicine (ethics no. DF2020-069-01). Written informed consent was obtained from the studied participants.

### 2.2. Purification of Serum Immunoglobulin

These serum samples were prepared according to the standard protocol. In brief, 5 mL of whole blood was collected from each individual and placed in a collection tube without an anticoagulant. After centrifugation at 2000 × g in a refrigerated centrifuge, these serum samples were then transferred to clean tubes and stored at −70°C [10]. Immunoglobulins from healthy donors, T1DM patients, and T2DM patients (*n* = 4, mixed sample) were isolated and purified using the commercial protein G 4FF prepacked chromatographic column from Sangon Biotech (Shanghai, China), following the manufacturer's instructions to purify the immunoglobulin from serum [11, 12]. These purified immunoglobulins were used in subsequent experiments.

### 2.3. Phage Titer Measurement

The M13 phage display peptide library was bought from New England Biolab (USA), and ER2738 *E.coli* (Thermo Fisher, USA) was incubated to an OD600 reading of 0.5. 200 *μ*L of ER2738 *E.coli* and 10 *μ*L of phage were added into each microcentrifuge tube, mixed, and incubated for 10 min at room temperature. The mixture was then transferred to preheated top agar (45°C) and mixed. The top agar was then poured into preheated LB/IPTG (Sangon Biotech, China) plates (37°C). The agar and plates were preheated to prevent unwanted solidification. The plates were then cooled down to room temperature for phage incubation overnight at 37°C. After overnight incubation, the blueish phage colonies were visible on the LB/IPTG media.

### 2.4. Biopanning Experiments

To select epitopes that can specifically bind to T1DM-associated antibodies in T1DM patients' serum, the purified serum immunoglobulin from healthy donors, T2DM patients, and T1DM patients were mixed and used as the base panning to coat 12 wells for each group (150 *μ*L for each well) in a 96-well plate. The plate was then shaken overnight at 4°C. 150 *μ*L of BSA (Sangon Biotech, China) was added into the test wells and TBST (TBS + 0.1% Tween-20) was used to wash the plate. The random phage library was diluted by TBST for 100 times. 150 *μ*L of the dilution was added into the first row and reacted with the serum immunoglobulin from healthy donors for 30 min. The unbonded phages were collected by a pipette and transferred to the second row containing T2DM immunoglobulin and cultured for another 30 min. The unbonded phages were finally collected by a pipette and transferred to the third row of T1DM immunoglobulin.

After shaking the plate for 30 min at room temperature, the unbonded phages were discarded by washing the wells of the third row with 100 *μ*L of TBST for 10 times. To collect the successfully bonded phages with the T1DM-associated immunoglobulin in the third row, 100 *μ*L of wash buffer (0.2 M) and glycine–HCl ((pH 2.2) 1 mg/mL BSA) (Sangon Biotech, China) were added into this row and incubated for 10 min to free the phages. The wash buffer from each well was then collected and neutralized with 150 *μ*L of 1 M Tris–HCl (pH=9.1). Collected phages with neutralized buffer were then added to 20 mL of ER2738 *E.coli* (OD600 = 0.01–0.05) and grew for 4.5 h at 37°C. 80% of the supernatant was collected and 2.7 mL of 20% PEG/2.5 M NaCl (Sangon Biotech, China) was added to each tube. The mixture was stored at 4°C overnight.

After spinning the centrifuge tubes, the supernatant was discarded. The phages should be visible on the walls of the centrifuge tubes. 1 mL of TBS was used to resuspend the phage and mixed with 133 *μ*L of 20% PEG/2.5 M NaCl. The microcentrifuge tubes were then iced for an hour and spun under 14,000 rpm at 4°C for 10 min. The remaining phages were then resuspended with 200 *μ*L of TBS. The collected phages were then titrated and used for another round of panning. After completing four rounds of panning, we titrated the phages. Finally, 1200 phage clones were selected after three times of biopanning. The above phage clone is subjected to amplification for sequencing.

### 2.5. Phage Amplification and DNA Extraction

To obtain monoclonal phages, toothpicks were used to collect phage colonies from the plates in phage titration. Monoclonal phage colonies were then incubated in 2 mL of ER2738 *E.coli* for 4 h. Culture media was transferred into microcentrifuge tubes and spun at 14000 rpm for 30 min at 4°C. 500 *μ*L of the culture supernatant was transferred into new centrifuge tubes. 200 *μ*L of 20% PEG/2.5 M NaCl was added into each tube for 15 min incubation. Next, the tubes were spun at 14000 rmp for 10 min at 4°C. The phages remained in the microcentrifuge tubes might be visible at this time.

The microcentrifuge tubes were spun again and a pipette was used to remove as much supernatant as possible. 100 *μ*L of iodine buffer and 250 *μ*L of 100% ethanol (Sangon Biotech, China) were added into each tube to resuspend the phages. After a short period of culture, tubes were spun at 10,000 rpm for 10 min at 4°C, and the supernatant was then removed. Next, the remaining substance was washed with 100 *μ*L of 75% ethanol and then air-dried for 10 min. Finally, the phage DNAs were resuspended in 30 *μ*L of DEPC water, and using −96 g III sequencing primer (5′-HOCCC TCA TAG TTA GCG TAA CG-3′) for sequencing, DNA sequencing of this experiment was performed by Sangon Ltd. (Shanghai, China).

### 2.6. ELISA Detection of Phage With Epitope Sequence

The purified IgG of T1DM was initially incubated with the coating solution at 4°C overnight. After washing the plate, phages with different epitope sequences were added, and each group of phages was diluted in a four-fold gradient and incubated with the coated IgG at 37°C for 1 h. Then, after washing the plate three times, the HRP-labeled anti-M13 antibody (Sino Biological, China) was added and incubated at 37°C for 30 min. After washing the plate three times, the TMB reagent (Sangon Biotech, China) was added and incubated in the dark at room temperature for 15 min. Finally, the reaction was stopped by a stop solution (Sino Biological, China) to measure the absorbance at 450 nm in the ELISA reader (Tecan, Switzerland).

### 2.7. Prokaryotic Expression of Recombinant Protein

We performed a prokaryotic expression of a synthetic protein containing the above homologous sequence for the selected amino acid sequences of the islet amyloid polypeptide (IAPP) and zinc transporter 8 (ZNT8) homologous 7 peptides, and the above polypeptide sequences were initially extended to 50 amino acids to the carboxy terminus. At the same time, the carboxy terminus of the His-tag was terminated with 3 glutamines (Gln), and the recombinant plasmid was constructed using pTrHis2C (Miaoling Biology, China) as an expression vector, and then expressed in the BL21 (DE3) host strain (Tiangen, China). After the expression, isolation, and purification of the above recombinant protein, clinically derived serum samples were detected by ELISA using the harvested recombinant protein.

### 2.8. Analysis of Antibody-Binding Activities of Purified Recombinant Protein by ELISA

A 96-well reaction plate was coated with the two recombinant proteins as antigens which were expressed from the step above at 4°C overnight. The plate was washed, blocked, and incubated at room temperature for 1 h. After washing, 100 *μ*L of serum derived from healthy donors (*n* = 200), T1DM (*n* = 100), and T2DM (*n* = 200) were added to appropriate wells for conjunction. Following incubating and washing, the HRP-labeled mouse anti-human IgG was added (Sino Biological, China) as a detection antibody for 1 hour. After the reaction, the substrate was added after washing and the reaction was stopped by a stop solution (Sino Biological, China) to measure the absorbance at 450 nm in the ELISA reader (Tecan, Switzerland).

### 2.9. Statistical Analysis

Data from this experiment were expressed as standard error of the mean (± SD). The differences between multiple groups were evaluated by one-way ANOVA. For all tests, *p*  <  0.05 for the difference was statistically significant (⁣^∗^*p*  <  0.05, ⁣^∗∗^*p*  <  0.01, and ⁣^∗∗∗∗^*p*  <  0.0001). All statistical analyses were performed using GraphPad Prism 8.3.0.

## 3. Results

### 3.1. Separation and Purification of Serum Immunoglobulin

To eliminate the influence of other components in serum on the screening of protein-peptide epitopes, we performed a protein G affinity chromatography on the collected clinical source serum and then passed the serum components and purified immunoglobulin on SDS–PAGE gel. PAGE results showed that after purification by affinity chromatography, most of the serum components can be separated and removed to retain immunoglobulin. The light and heavy chains of immunoglobulin can be clearly observed, among which the heavy chain's molecular weight is about 50 KD and the molecular weight of the light chain is about 25 KD (Figure S1).

### 3.2. Enrichment of Phage-Displayed IgG-Binding Peptides by Patient IgG

First, the M13 phage display peptide library was diluted and their titer was determined. A single blue plaque could be observed at 2.0 × 10^10^ pfu (Figure S2). The titer was then used for biopanning. After each round of enrichment, the eluted phage was infected with ER2738 *E.coli*, and the phage titer was determined on the LB/IPTG media. A 31.6111.1 and 72.4-fold increase in the ratio of phage particles eluted to the phage particles applied for enrichment was noted after the fourth round and three times of biopanning indicating enrichment of the phage display library (Supporting Table 1).

### 3.3. Elutriated Peptide Sequence Sequencing Results

In the previous experiment, 1200 monoclonal phages were selected for DNA sequencing, and a total of 1077 phages were finally passed to sequencing. Accounting for 89.75% of the total samples, 93 epitope sequences were selected. After excluding repeated sequences, 73 sequences were obtained, Moreover, 9 sequences with a high frequency of representation were finally sequenced (Supporting Table 2).

By analyzing the sequencing results, we found 9 specific homologous protein sequences associated with T1DM. The sequences of them were checked by “Protein Blast” (https://www.ncbi.nlm.nih.gov/). Two of them were selected for our experiment. The “AVEVLKR” polypeptide sequence was detected in 88 phage clones, and it was found to be the homologous sequence of human IAPP. The “RLTFGWH” homologous polypeptide sequence was detected in 44 phage clones, and this homologous sequence was found to be derived from human ZNT8. Except these two sequences, the remaining 7 polypeptide sequences were derived from nonhuman proteins and are associated with certain exogenous protein antigen components (Table 2).

### 3.4. The Results of ELISA Binding Test Between Epitope and IgG of T1DM

The IgG of T1DM was coated on the plate for ELISA experiments to study the binding of epitopes to IgG. The results are shown in Table 3. These selected nine epitope sequences bound well to T1DM-derived IgG, and the absorbance at 450 nm was mostly much larger than that of the control group. The nine epitope sequences bound to IgG in a dose-dependent relationship.

### 3.5. Identification of Target Protein of Prokaryotic Expression

IAPP and ZNT8 as target genes form fusion plasmids with pTrHis2C, and the map of pTrHis2C could be seen in Figure S3. The recombinant proteins IAPP and ZNT8 were expressed by prokaryotic cells BL21 (DE3) host strain. The molecular weights of the recombinant proteins IAPP and ZNT8 were 9 and 17 kDA, respectively, which were displayed in the SDS–PAGE (Figure S4).

### 3.6. The Antigen Specificity of the Expressed Recombinant Protein Was Detected by ELISA

The two purified recombinant proteins (IAPP and ZNT8) were coated on a 96-well plate as antigens. The specific reaction between antigens and the serum of healthy (*n* = 200) donors, T2DM (*n* = 200), and T1DM (*n* = 100) was detected by direct ELISA.

**Figure 1:**
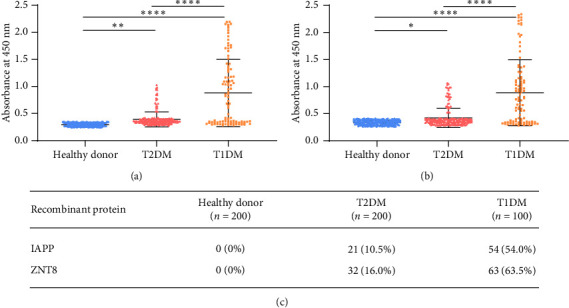
Comparison of reactivity between the recombinant antigen and clinical samples. (a) The absorbance of IAPP at 450 nm in different samples. (b) The absorbance of ZNT8 at 450 nm in different samples. (c) Positive rate detection of IAPP and ZNT8. Data are presented as mean ± SD. The significance level was selected as 0.05. ⁣^∗^*p* < 0.05, ⁣^∗∗^*p* < 0.01, and ⁣^∗∗∗∗^*p* < 0.0001.

To be specific, the healthy donor showed negative results for IAPP antigen, and the two groups of patients had a significantly high level of absorbance at 450 nm compared to the healthy donor group. Moreover, the absorbance of the T1DM (*n* = 100) group was significantly higher than that of the T2DM group (*n* = 200) (Figure 1(a)). There were 21 positive results in T2DM, whose positive rate was 10.5% and 54 positive results were observed in T2DM samples, whose positive detection rate was 54.0% (Figure 1(c)). Similar to the results of IAPP, the absorbance of healthy donors of ZNT8 was significantly lower than that of the patient group, and the absorbance level of the T1DM group was also higher than the T2DM group (Figure 1(b)). There were 32 positive results in T2DM, whose positive rate was 16.0%, and 63 positive results were detected in T1DM, whose positive rate was 63.0% (Figure 1(c)). These results indicated that the recombinant proteins IAPP and ZNT8 could specifically bind to the serum IgG of T1DM, even if there was also a small amount of binding in the samples of T2DM.

## 4. Discussion

Commercialized phage display systems have been widely used in various scientific studies, especially for the study of interactions between proteins and proteins, including antigen-antibody reactions, enzyme and substrate reactions, and cytokine and cell surface receptors [13]. In this study, we analyzed T1DM-specific autoantibody epitope studies using NEB's phage display method. In accordance with the kit's instructions, we used the phage library Ph.D.-7 to contain a random 7 amino acid phage envelope protein in sequence with normal serum, T2DM serum, and T1DM serum. A total of 9 conserved 7-peptide amino acid sequences were obtained after the sequencing analysis of 1200 phage clones. By protein BLAST search alignment, it was observed that the “AVEVLKR” and “RLTFGWH” 7-peptide sequences have identical homology with IAPP and ZNT8, respectively. Both of them in our results show a close connection to T1DM. Among these two polypeptides, the ZNT8 was confirmed to be closely related to T1DM [14–16]. The specific autoantigen, the epitope of the autoantigen, is likely to be in the 7-peptide structure of “RLTFGWH.”

The aforementioned findings also revealed a greater concentration of ZNT8 autoantibodies in the serum of T1DM patients. Therefore, finding ZNT8 autoantibodies in serum is very important for determining the presence of T1DM (this finding suggests that ZNT8 may someday be employed as a novel biomarker to identify T1DM). On the other hand, we discovered that patients with T1DM have autoantibodies against IAPP in their serum. The positive rate of ZNT8 autoantibody detection in the T1DM sample is 63% and 16% for T2DM. As T1DM and ZNT8-autoantibody are strongly connected, in the future, ZNT8-autoantibody can be used as an essential marker molecule for type I diabetes diagnosis.

ZNT8 is mainly responsible for zinc enrichment in insulin-secreting granules, and the increase of zinc accumulation in *β*-cells is the result of ZNT8 overexpression [17], as it acts as a major target of CD8 T cells and is attacked by the autoimmune system [18]. Similar to the existing research results, our above results also showed that there is a higher level of ZNT8 autoantibodies in the serum of patients with T1DM, and we found the specific epitope sequence information binding to autoantibodies. Therefore, the detection of ZNT8 autoantibodies in serum is of great value for the diagnosis of T1DM, and this result indicates that ZNT8 could be potentially used as a novel biomarker to diagnose T1DM [19]. Also, our study found that there are autoantibodies against IAPP in the serum of patients with T1DM. The positive rate of IAPP detection in the T1DM sample is 54% and 10.5% for T2DM. IAPP has been extensively studied in T2DM [20], although some research has attempted to link IAPP with T1DM, no specific evidence has been found. These results indicate the correlation between IAPP and T1DM and also suggest that the IAPP autoantibody could be used for accurate diagnosis of T1DM.

In this study, another important finding was the successive detection of two different 7-peptide sequences “HWNTVVS” and “MPSATPP” and the AAA family ATPase of d-proteobacteria and SDR family NAD (P)-dependent oxidoreductases of b-proteobacteria, respectively. With complete homology, the results showed that the human serum of T1DM is rich in antibodies specific for Proteus species compared with healthy donors and T2DM patients. Proteus species are normal flora of the intestine and are involved in the construction of the intestinal microecological environment [21]. Proteus species in the intestine of healthy people do not cause an infection like a normal parasite and cannot stimulate the immune system to produce corresponding antibodies [22]. However, in this study, we found that patients with T1DM had a characteristic higher titer of antibodies against Proteus ATPase and oxidoreductase compared with the serum of healthy individuals and T2DM patients. Diabetes patients are often accompanied by opportunistic infections [23], therefore, the prevalence of microbe infection in the serum of diabetic patients is understandable. However, compared with T2DM, why T1DM has such a specific antiprotein-specific antigen antibodies' profile remains a question. The antigenic components of microorganisms may bear resemblance to host epitopes, potentially triggering an autoimmune reaction, such as in the case of acute rheumatic fever. Moreover, the composition of microflora could impact the susceptibility to autoimmunity [24]. Therefore, the finding of Proteus infection may relate to the pathogenic process of T1DM.

Meanwhile, another polypeptide sequence “HGLLSML” is derived from the ABC transporter ATP-binding protein of *Kitasatospora acidiphila*, which seems to have a function of energy transmission when the bacteria or other microbes accompany T1DM. Bacteria employ various transport proteins, such as ATP-binding cassette (ABC) transporters, to obtain vital nutrients from the host and manage the impact of toxicity. The energy obtained from ATP hydrolysis is utilized to move the substrate across the membrane against a concentration gradient [25]. This is also a potential biomarker of bacterial infection and pathogenesis.

To accurately diagnose and classify diabetes, future studies need to focus on detecting antibodies against these energy-related proteins, as these studies have feasibility and clinical value. Another striking finding is that in addition to the presence of ATPases against the Proteus AAA family in the serum of patients with T1DM, we also found that the polypeptide epitope “SLIAHPQ” screened by the phage display system is derived from the AAA family ATPase of actinomycetes. Interestingly, this result indicates that their ATPase antibodies are not only derived from Proteus but also from other bacterial sources in the serum of patients with T1DM compared with healthy donors' serum and T2DM patients. These findings suggest that the infected bacteria associated with T1DM may have unique energy metabolism processes and consequences, resulting in specific immune responses and immune effector molecules.

The findings of this study also demonstrate that several other bacterial and fungal protein components can be detected in T1DM serum, such as the polypeptide sequence “IVTQIPM” has been identified as the exogenous RND penetrating the enzyme subunit of *Porphyromonas gingivalis*. It has been confirmed to cause periodontal disease complications of T1DM [26]. The “QTWSHVQ” common amino acid sequence can be confirmed to have homology with an unidentified protease from the genus Cyanobacteria. In contrast to healthy people and T2DM patients, the above results suggest that T1DM is associated with particular microbial infections and microecological changes, especially for energy-metabolizing molecules of certain microorganisms. Immune effector molecules are produced as a result of the immune system's robust immunological response. However, more investigation and testing are needed to determine whether these immune responses play a role in the pathological process of the islet cells in T1DM; as well as whether the characteristic antibodies to these metabolism-related molecules and other bacterial metabolites can be used to accurately diagnose and classify diabetes require further research and evaluation.

There are still some limitations in this study. The phage display system revealed nine protein sequences specific to T1DM. In Figure 1, T2DM serum (autoantibody) reacts with recombinant IAPP and ZNT8 proteins. However, we further detect it by recombinant proteins of these two peptides that people who have diabetes may have autoantibodies against sequences of IAPP and ZNT8 other than those found in the phage display system. The specificity of the screening system seems to depend on the phage library. Moreover, the phage display may cause false positive hits due to wrong binding on nontarget-related materials used during the selection. Therefore, the library selection and the experiment for further study must be performed under careful control.

Phage display systems have been demonstrated in numerous research, including this one, to be a crucial tool for the investigation of diabetes-associated autoantibody spectrum. This research tool can not only be used to find the characteristic antibody spectrum and antibody epitopes of different types of diabetes but also to study the different microecological components and accompanying patients of diabetes as well. Thus, further research and attention should be given to the characteristics of microbial infection shown in this study.

## 5. Conclusion

The phage display technique is a powerful and reliable method for researching high-throughput antigen-antibody reactions. In this study, human pancreatic and concurrent bacterial-derived protein antigens and their epitopes were identified, which is crucial for distinguishing different types of diabetes. It might serve as a crucial indicator for diabetes diagnosis in the future.

## Figures and Tables

**Table 1:** Demographics of healthy and diabetic subjects.

	Healthy donor (*n* = 200)	T1DM (*n* = 100)	T2DM (*n* = 200)	*p* value
Age (years)	31.1 ± 9.7	36.9 ± 15.6	39.7 ± 17.1	0.027
Gender (female %)	108 (54)	63 (63)	114 (57)	0.759
BMI	21.8 ± 3.5	16.8 ± 3.1	23.1 ± 5.4	0.016
Family history (yes %)	—	30 (30)	67 (33.5)	0.344
Age at onset	—	19 ± 8	27 ± 6	< 0.01
FPG	4.98 ± 0.67	18.32 ± 12.58	12.41 ± 7.32	< 0.01
BUN	5.72 ± 1.32	10.12 ± 4.48	7.23 ± 4.14	< 0.01
CR	87.13 ± 20.88	110.21 ± 32.27	99.21 ± 54.47	< 0.01
UA	296.87 ± 101.57	301.92 ± 97.14	321.72 ± 12.21	0.582
HbA1C (%)	5.14 ± 1.72	7.31 ± 3.35	8.42 ± 4.14	< 0.01
TG	1.08 ± 0.45	1.24 ± 1.12	1.68 ± 1.33	0.036
HDL-c	1.67 ± 0.41	1.56 ± 0.21	1.02 ± 0.45	0.041
LDL-c	3.31 ± 0.55	3.31 ± 1.25	4.27 ± 1.39	0.012

**Table 2:** The homologous epitope peptide sequence found in this study.

Phage clones	Peptide sequence	Same peptide sequence frequency	Homology protein	Query cover (%)
GZ1804240135 et al	AVEVLKR	88	Islet amyloid polypeptide isoform X1	100
GZ18042401010 et al	RLTFGWH	44	Zinc transporter 8	100
GZ18042400971 et al	HWNTVVS	75	AAA family ATPase	100
GZ18062901033 et al	HGLLSML	52	ABC transporter ATP-binding protein	100
GZ18042400979 et al	IVTQIPM	36	Efflux RND transporter permease subunit	100
GZ18050801683 et al	MPSATPP	25	SDR family NAD (P)-dependent oxidoreductase	100
GZ18062700708 et al	QTWSHVQ	33	Hypothetical protein	100
GZ18062700720 et al	FYGNSDA	33	YSIRK-type signal peptide-containing protein	100
GZ18011101884 et al	SLIAHPQ	8	AAA family ATPase	100

*Note:* Same peptide sequence frequency refers to the frequency of the same random seven-peptide sequence appearing in different screening phages.

**Table 3:** Analysis of antigen peptide binding activities of IgG according to ELISA.

Phage titer (pfu)	Peptide sequence									
	AVEVLKR	RLTFGWH	HWNTVVS	HGLLSML	IVTQIPM	MPSATPP	QTWSHVQ	FYGNSDA	SLIAHPQ	Blank
1.0 × 1012	2.688	4.897	2.287	3.866	2.134	1.895	2.725	2.849	1.073	0.340
2.5 × 1011	3.071	4.827	1.763	3.509	2.568	1.875	2.222	2.911	0.895	0.156
6.25 × 1010	2.015	5.198	1.864	3.590	2.758	1.233	3.000	2.347	0.769	0.083
1.56 × 1010	2.276	4.259	0.835	2.683	1.890	1.054	2.011	2.408	0.350	0.066
3.9 × 109	1.834	3.783	0.578	1.965	1.653	0.879	1.883	2.198	0.132	0.052
9.7 × 108	0.866	1.945	0.145	1.327	0.256	0.345	1.407	0.894	0.033	0.011
2.4 × 108	0.134	0.954	0.095	1.338	0.342	0.009	1.006	1.657	0.188	0.017
6 × 107	0.040	0.453	0.045	0.761	0.055	0.128	0.792	1.175	0.109	0.004
1.5 × 107	0.060	0.144	0.003	0.458	0.008	0.023	0.483	0.567	0.099	0.005
3.75 × 106	0.128	0.032	0.007	0.231	0.003	0.007	0.286	0.258	0.102	0.006
9.375 × 105	0.103	0.052	0.009	0.346	0.010	0.002	0.124	0.034	0.133	0.003
2.34 × 105	0.003	0.001	0.012	0.001	0.081	0.005	0.030	0.004	0.100	0.002

## Data Availability

The clinical data and phage display results used to support the findings of this study are included within the article. Please check Tables 1, 2, 3, supporting table 1, and supporting table 2 for data detail. Other information and data to support the findings of this study are included within the Supporting Information files.

## Supporting Information

Supporting Information Figure S1: Results of SDS–PAGE after purification of serum immunoglobulin by protein G affinity chromatography.

Supporting Information Figure S2: Single blue plaque checking on agar gel.

Supporting Information Figure S3: Plasmid pTrHis2C map.

Supporting Information Figure S4: Detection of recombinant protein expression.

Supporting Information Supporting Table 1: Analysis of times and rounds of biopanning of the phage display library with T1DM IgG.

Supporting Information Supporting Table 2: Statistics of biopanning peptide sequences.

## References

[1] Ilonen J., Lempainen J., Veijola R. (2019). The Heterogeneous Pathogenesis of Type 1 Diabetes Mellitus. *Nature Reviews Endocrinology*.

[2] Syed F. Z. (2022). Type 1 Diabetes Mellitus. *Annals of Internal Medicine*.

[3] Li W., Huang E., Gao S. (2017). Type 1 Diabetes Mellitus and Cognitive Impairments: A Systematic Review. *Journal of Alzheimer’s Disease*.

[4] Yang Y., Day J., Souza-Fonseca Guimaraes F., Wicks I. P., Louis C. (2021). Natural Killer Cells in Inflammatory Autoimmune Diseases. *Clinical and Translational Immunology*.

[5] Das A. K. (2015). Type 1 Diabetes in India: Overall Insights. *Indian Journal of Endocrinology and Metabolism*.

[6] Michels A. W., von Herrath M. (2011). Update: Antigen-Specific Therapy in Type 1 Diabetes. *Current Opinion in Endocrinology Diabetes and Obesity*.

[7] Nagano K., Tsutsumi Y. (2021). Phage Display Technology as a Powerful Platform for Antibody Drug Discovery. *Viruses*.

[8] Jespers L. S., Messens J. H., Keyser A. D. (1995). Surface Expression and Ligand-Based Selection of cDNAs Fused to Filamentous Phage Gene VI. *Nature Biotechnology*.

[9] Pande J., Szewczyk M. M., Grover A. K. (2010). Phage Display: Concept, Innovations, Applications and Future. *Biotechnology Advances*.

[10] Wu F. L., Lai D. Y., Ding H. H. (2019). Identification of Serum Biomarkers for Systemic Lupus Erythematosus Using a Library of Phage Displayed Random Peptides and Deep Sequencing. *Molecular & Cellular Proteomics*.

[11] Andrew S. M., Titus J. A. (2001). Purification of Immunoglobulin G. *Current Protocols in Cell Biology*.

[12] Cooper H. M., Paterson Y. (2001). Purification of Immunoglobulin G Fraction from Antiserum, Ascites Fluid, or Hybridoma Supernatant. *Current Protocols in Molecular Biology*.

[13] Pierzynowska K., Morcinek-Orłowska J., Gaffke L., Jaroszewicz W., Skowron P. M., Węgrzyn G. (2023). Applications of the Phage Display Technology in Molecular Biology, Biotechnology and Medicine. *Critical Reviews in Microbiology*.

[14] Mao Z., Lin H., Su W. (2019). Deficiency of ZnT8 Promotes Adiposity and Metabolic Dysfunction by Increasing Peripheral Serotonin Production. *Diabetes*.

[15] Pociot F., Lernmark Å. (2016). Genetic Risk Factors for Type 1 Diabetes. *The Lancet*.

[16] Howson J. M., Krause S., Stevens H. (2012). Genetic Association of Zinc Transporter 8 (ZnT8) Autoantibodies in Type 1 Diabetes Cases. *Diabetologia*.

[17] Kleiner S., Gomez D., Megra B. (2018). Mice Harboring the Human SLC30A8 R138X Loss-Of-Function Mutation Have Increased Insulin Secretory Capacity. *Proceedings of the National Academy of Sciences of the United States of America*.

[18] Énée É., Kratzer R., Arnoux J. B. (2012). ZnT8 is a Major CD8+ T Cell-Recognized Autoantigen in Pediatric Type 1 Diabetes. *Diabetes*.

[19] Zhang X., Dong Y., Liu D., Yang L., Xu J., Wang Q. (2022). Antigen-Specific Immunotherapies in Type 1 Diabetes. *Journal of Trace Elements in Medicine and Biology*.

[20] Zhang G., Meng L., Wang Z. (2022). Islet Amyloid Polypeptide Cross-Seeds Tau and Drives the Neurofibrillary Pathology in Alzheimer’s Disease. *Molecular Neurodegeneration*.

[21] Hamilton A. L., Kamm M. A., Ng S. C., Morrison M. (2018). Proteus spp. as Putative Gastrointestinal Pathogens. *Clinical Microbiology Reviews*.

[22] Drzewiecka D. (2016). Significance and Roles of Proteus spp. Bacteria in Natural Environments. *Microbial Ecology*.

[23] Qin J., Li Y., Cai Z. (2012). A Metagenome-wide Association Study of Gut Microbiota in Type 2 Diabetes. *Nature*.

[24] Wang L., Wang F. S., Gershwin M. E. (2015). Human Autoimmune Diseases: A Comprehensive Update. *Journal of Internal Medicine*.

[25] Tanaka K. J., Song S., Mason K., Pinkett H. W. (2018). Selective Substrate Uptake: The Role of ATP-Binding Cassette (ABC) Importers in Pathogenesis. *Biochimica et Biophysica Acta (BBA)-Biomembranes*.

[26] Lappin D. F., Robertson D., Hodge P. (2015). The Influence of Glycated Hemoglobin on the Cross Susceptibility Between Type 1 Diabetes Mellitus and Periodontal Disease. *Journal of Periodontology*.

[27] Yuan Ji, Wang Z., Wang H. (2023). *Screening of Diabetes-Associated Autoantigens and Serum Antibody Profiles by Phage Display System*.

