# Supplementary material for: Screening of Diabetes-Associated Autoantigens and Serum Antibody Profiles Using a Phage Display System
Source: Int J Microbiol. 2024 Oct 24;2024:1220644. doi: 10.1155/2024/1220644 (PMC11527542; doi:10.1155/2024/1220644)
Supplement: Supporting Information — Figure S1: Results of SDS–PAGE after purification of serum immunoglobulin by protein G affinity chromatography. [file 1220644.f1.docx]

**SUPPLEMENTARY**

**FIGURES**


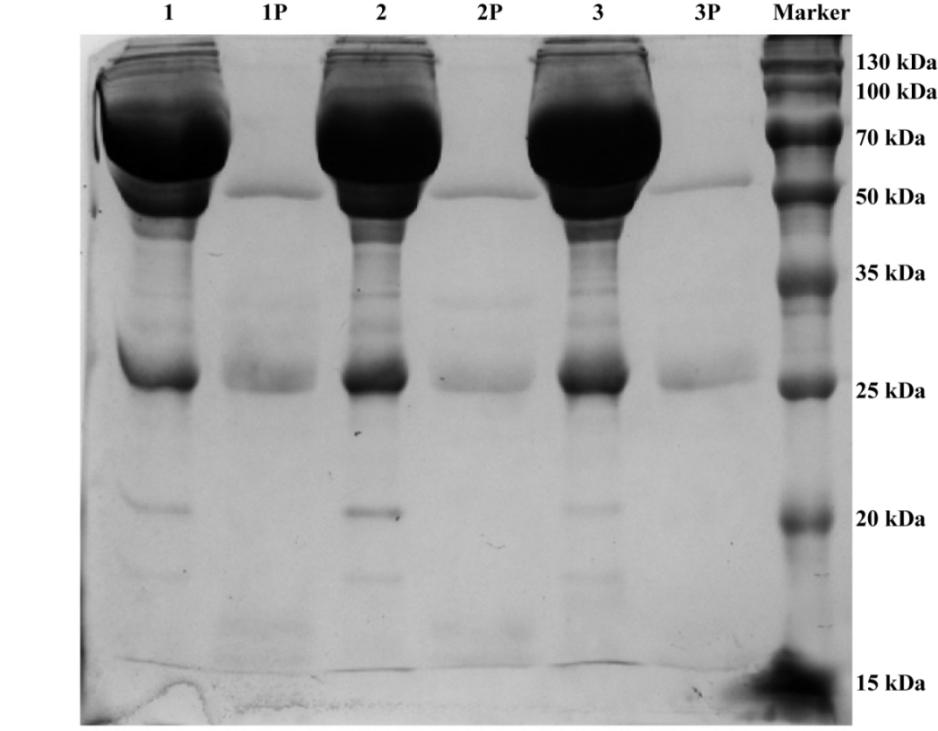


**Figure S1. Results of SDS-PAGE electrophoresis after purification of serum immunoglobulin by protein G affinity chromatography.** Column 1, 2, 3 are the original serum; 1P, 2P and 3P are the purified serum immunoglobulins.


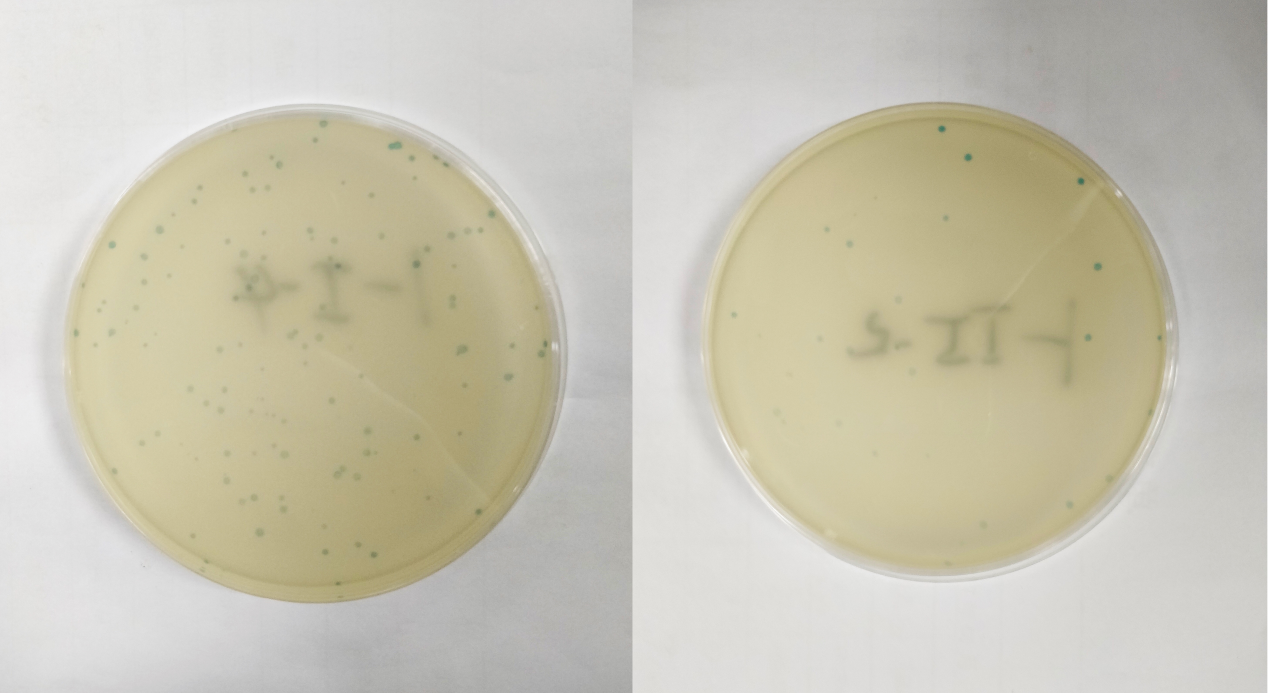


**Figure S2.** **Single blue plaque checking on agar gel.** The blue plaque was clearly visible at the appropriate titer level.


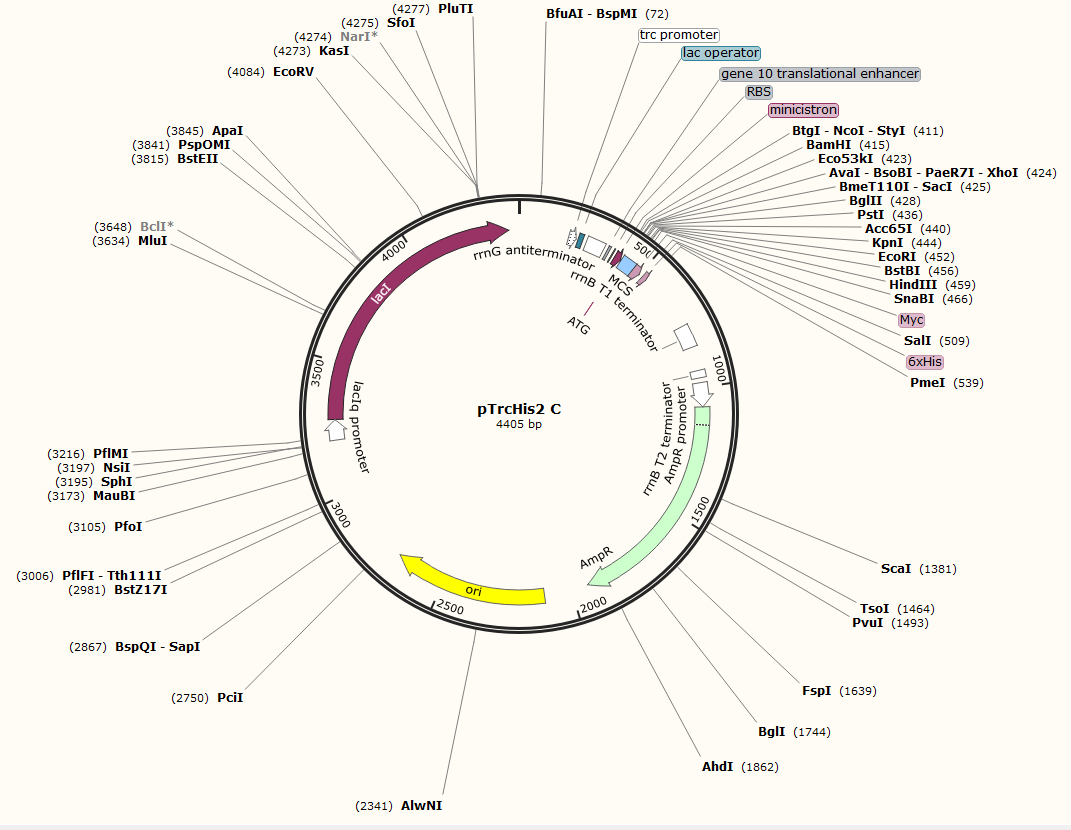


**Figure S3.** **Plasmid pTrcHis2 C map (download from https://www.snapgene.com/).**


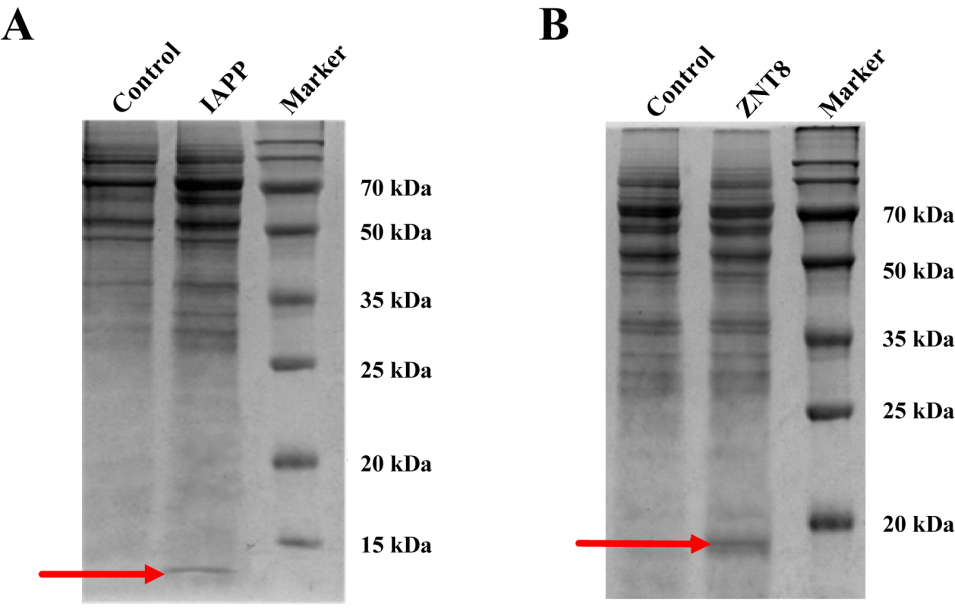


**Figure S4. Detection of recombinant protein expression.**(A) SDS-PAGE analysis of IAPP. (B) SDS-PAGE analysis of ZNT8. The red arrow points to the target protein.

**TABLES**

**Supplementary Table 1. Analysis of times and rounds of bio-panning of the phage display library with T1DM IgG.**

pfu: plaque forming unit

**Supplementary Table 2. Statistics of biopanning peptide sequences**
